# The Diagnostic Accuracy of Truncal Ataxia and HINTS as Cardinal Signs for Acute Vestibular Syndrome

**DOI:** 10.3389/fneur.2016.00125

**Published:** 2016-08-08

**Authors:** Sergio Carmona, Carlos Martínez, Guillermo Zalazar, Marcela Moro, Angel Batuecas-Caletrio, Leonel Luis, Carlos Gordon

**Affiliations:** ^1^Fundación San Lucas, Rosario, Argentina; ^2^Hospital José María Cullen, Santa Fe, Argentina; ^3^Unidad de Otoneurología, Servicio de Otorrinolaringología y Patología Máxilofacial, Hospital Universitario de Salamanca, Salamanca, Spain; ^4^Translational Clinical Physiology Unit, Faculty of Medicine, Institute of Molecular Medicine, University of Lisbon, Lisbon, Portugal; ^5^Otolaryngology Unit, Department of Surgical Specialities and Anesthesiology, Hospital de Cascais, Lisbon, Portugal; ^6^Department of Neurology, Meir Medical Center, Kfar-Saba, Israel; ^7^Sackler Faculty of Medicine, Tel Aviv University, Tel Aviv, Israel

**Keywords:** AVS, hints, truncal ataxia, AICA, pica, vestibular neuritis

## Abstract

The head impulse, nystagmus type, test of skew (HINTS) protocol set a new paradigm to differentiate peripheral vestibular disease from stroke in patients with acute vestibular syndrome (AVS). The relationship between degree of truncal ataxia and stroke has not been systematically studied in patients with AVS. We studied a group of 114 patients who were admitted to a General Hospital due to AVS, 72 of them with vestibular neuritis (based on positive head impulse, abnormal caloric tests, and negative MRI) and the rest with stroke: 32 in the posterior inferior cerebellar artery (PICA) territory (positive HINTS findings, positive MRI) and 10 in the anterior inferior cerebellar artery (AICA) territory (variable findings and grade 3 ataxia, positive MRI). Truncal ataxia was measured by independent observers as grade 1, mild to moderate imbalance with walking independently; grade 2, severe imbalance with standing, but cannot walk without support; and grade 3, falling at upright posture. When we applied the HINTS protocol to our sample, we obtained 100% sensitivity and 94.4% specificity, similar to previously published findings. Only those patients with stroke presented with grade 3 ataxia. Of those with grade 2 ataxia (*n* = 38), 11 had cerebellar stroke and 28 had vestibular neuritis, not related to the patient’s age. Grade 2–3 ataxia was 92.9% sensitive and 61.1% specific to detect AICA/PICA stroke in patients with AVS, with 100% sensitivity to detect AICA stroke. In turn, two signs (nystagmus of central origin and grade 2–3 Ataxia) had 100% sensitivity and 61.1% specificity. Ataxia is less sensitive than HINTS but much easier to evaluate.

## Introduction

It is crucial to differentiate stroke from peripheral vestibular syndromes (PVSs), as this is essential when deciding how to treat the patient. In the case of stroke, the patient must be hospitalized and endovascular treatments may be chosen thus avoiding a posterior fossa edema, which may complicate the prognosis.

In 2006, Lee et al. described that the cerebellar infarction territory most commonly involved that simulated vestibular neuritis was the medial branch or the posterior inferior cerebellar artery (PICA) territory (96%), followed by the anterior inferior cerebellar artery (AICA) territory (4%) (1). In 2009, the head impulse, nystagmus type, test of skew (HINTS) protocol was designed to differentiate peripheral vestibular disease from stroke in patients with acute vestibular syndrome (AVS) (2). HINTS requires specific skills, which general physicians who treat patients first in the emergency room may not possess, and there is no easy, observational test, such as a truncal ataxia rating scale, to help with differential diagnosis of stroke in patients with AVS. The relationship between the degree of truncal ataxia and stroke has not been systematically studied in patients with AVS. Even though the importance of evaluating ataxia has been proposed by other authors (1, 3), none of them has systematically studied it. Its evaluation and quantification is much simpler than that of oculomotor signs.

The term AVS was initially introduced by Hotson and Baloh in 1998 (4). In 2006, the Classification Committee of the Bárány Society (CCBS) convened its first meeting to begin structuring the approach to developing the International Classification of Vestibular Disorders (ICVD). It was then established that “AVS comprises diseases and disorders that usually manifest themselves with a single episode of sudden onset of vestibular symptoms and signs: vomiting, nystagmus and postural instability” (5).

### Objectives

The objective of this work is to assess the degree of sensitivity and specificity of different isolated or combined signs in the differential diagnosis of AVS and to compare these findings with those reported in the HINTS protocol. Another objective is to assess the possibility of selecting signs that are easy to interpret when discriminating central causes of AVS as accurately as possible, and which are easy enough to be used by physicians on duty who do not have specific training in clinical neurology.

The purpose of this study is to determine the diagnostic accuracy of a truncal ataxia rating scale, compared to the HINTS protocol in the differential diagnosis of patients with AVS.

## Materials and Methods

Between 2006 and 2012, 114 patients with AVS were enrolled out of a group of 1218 patients who visited the emergency room at a high complexity reference hospital in the city of Santa Fe, Argentina (population 400,000 people) due to dizziness. A retrospective, observational, descriptive, and cross-sectional study was conducted. All the patients admitted signed an informed consent, and the research protocol was assessed by an Independent Ethics Committee of Cullen Hospital (dependant on Universidad Nacional del Litoral), as established by the World Medical Association Declaration of Helsinki.

The general neurological and otoneurological exams assessed were as follows: vestibulo–ocular reflex (VOR) static signs (spontaneous nystagmus, observation under Frenzel glasses); VOR dynamic signs (head impulse test, repeated 20 times for each side in a random order) (6); VOR cancelation; and test of skew deviation by means of cover cross-cover test (7, 8); Static and dynamic signs of vestibulo-spinal reflex (deviation in Bárány’s pointing test; Romberg sign; Fukuda stepping test; tandem gait), positional Dix–Hallpike and roll tests. These exams were performed in the emergency room by neurology residents who were blinded to the MRI and caloric test results. Within 24 h, patients were again evaluated by a neurologist who was not blinded and who confirmed the findings. As gold standard, all patients underwent the following tests: brain diffusion MRI angiography within 48 h of admission, but never within the first 12 h. The routine MRI at the hospital was performed with a 1-T General Electric Superconductive Resonator, using T1- and T2-weighted and diffusion-weighted imaging (DWI) and FLAIR. The results always agreed with the clinical findings, in some cases a high field MRI was performed with a 1.5-T (Phillips Gyroscan) outside the hospital, and the results showed no differences. Caloric tests were performed by the hospital neurotologist within the first 48 h, under video-Frenzel’s observation, using Cawthorne and Hallpike technique, and assessing duration of nystagmus in each ear. Pure tone audiograms were performed within the first 48–72 h by audiologists from the Audiology department using a Kamplex Interacoustic AA222 equipment. Hearing was also evaluated using fork tests.

Cerebellum function was assessed during the general neurological exam: index-nose test, heel-knee test, trunk coordination. Truncal ataxia was measured using independent observers as grade 1, mild to moderate imbalance with walking independently; grade 2, severe imbalance with standing, but cannot walk without support; grade 3, falling at upright posture (1). Additionally, the ability to sit up from supine without using the arms was assessed, a sign that Babinski described in 1913 as an indication of asynergy (9). This sign was considered positive when the patient was unable to do this. This sign can also be positive in case of hemiplegia, which none of our patients presented. Within 24 h of the patients’ admission, they were reevaluated by another member of the neurology team to check the grade of ataxia and the rest of the neurological exam.

The signs already evaluated in the HINTS (2) protocol, either in isolation or in combination, were used for statistical analysis. The usefulness of postural truncal ataxia was evaluated in isolation or in combination, as it is an easy sign to interpret even for physicians with little training in clinical neurology, and it has been suggested that it is an element to consider when discriminating the central from the peripheral origin of AVS (3, 10).

SPSS Statistics 17.0 software package was used for statistical analysis. In the case of continuous variables, distribution was evaluated first using Kolmogorov–Smirnov test, and a non-parametric test was used if there was no normal distribution in the Mann–Whitney *U*. In the case of categorical variables, Chi Square or Fisher’s exact test was used with Yates correction, if necessary.

## Results

**Figure 1:**
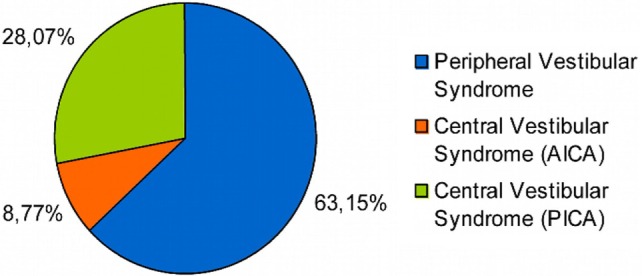
**Distribution of lesions in patients with AVS**.

Out of the 114 patients selected, in 72 of them the final diagnosis was vestibular neuritis and in 42 of them stroke, out of which 32 belonged to the PICA territory and 10 to the AICA one (Figure 1). There were no significant differences between patients with PVS and central vestibular syndrome (CVS) as regards to sex (*p* > 0.05), though there were statistically significant differences (*p* < 0.05) in variables: (1) age and (2) presence of cardiovascular risk factors (i.e., diabetes mellitus, hypertension, obesity, dyslipidemia, smoking, alcohol abuse) (Table 1).

**Table 1:** **Demographic characteristics**.

	Peripheral vestibular syndrome	Central vestibular syndrome	
Sex	F 44 M 28	F 22 M 20	NS^a^
Age	43.3 (±14.9)	57.9 (±11)	*p* < 0.05^b^
Diabetes mellitus	–	14	*p* < 0.05^b^
Hypertension	14	29	*p* < 0.05^b^
Obesity	1	11	*p* < 0.05^b^
Dyslipemia	1	13	*p* < 0.05^b^
Smoking	1	7	*p* < 0.05^b^
Alcohol abuse	–	3	*p* < 0.05^b^

*^a^Square Chi test and Fisher’s exact test depending on the case*.

*^b^Non-parametric test (Mann–Whitney test) was used when variable had not normal distribution evaluated using Kolmogorov–Smirnov test*.

Head impulse, nystagmus type, test of skew protocol signs and ataxia grade were evaluated. As shown in Table 2, the main findings were that HIT was always positive on the affected side in vestibular neuritis (72/72), always negative in PICA stroke (0/32), but positive in most of AICA cases (7/10), and that grade 3 ataxia was never present in vestibular neuritis (0/72). All patients with grade 3 ataxia had a positive Babinski’s asynergy sign (see Video S1 in Supplementary Material, PICA, truncal ataxia), but this sign was also present in three patients with stroke who had grade 1 ataxia and in seven of the patients with stroke with grade 2 ataxia.

**Table 2:** **Presence of evaluated signs in the different groups**.

	Central vestibular syndrome due to PICA lesion	Central vestibular syndrome due to AICA lesion	Peripheral vestibular syndrome (vestibular neuritis)
Direction-changing nystagmus	20/32	4/10	0/72
Unidirectional nystagmus	12/32	6/10	72/72
No ataxia	0/32	0/10	5/72
Grade 1 ataxia	3/32	0/10	39/72
Grade 2 ataxia	9/32	2/10	28/72
Grade 3 ataxia	20/32	8/10	0/72
Skew deviation+	17/32	6/10	4/72
HIT+	0/32	7/10	72/72

Table 3 shows the sensitivity and specificity of each evaluated sign; sensitivity is not very high in most signs when taken in isolation, and HIT and presence of grade 2–3 ataxia are the ones that show higher sensitivity, 83 and 92.9%, respectively. On the other hand, the specificity in some of these signs reaches 100% (i.e., nystagmus, HIT, grade 3 ataxia). If only grade 3 ataxia is considered in isolation, there was 66.7% sensitivity with 100% specificity for stroke in general (AICA and PICA lesion).

**Table 3:** **Sensitivity and specificity of each and combined sign**.

	Sensitivity (MRI+) (%)	Specificity (MRI−) (%)
Direction-changing nystagmus	57	100
Skew	54.8	94.4
HIT	83	100
Ataxia 1^a^	7.1	45.8
Ataxia 2^b^	26.2	61.1
Ataxia 3^c^	66.7	100
Ataxia 2–3	92.2	61.1
Asynergy+	92.9	100
HINTS	100	94.4
Ataxia 2–3 + direction-changing nystagmus	100	61.1
Ataxia 2–3 + skew	100	56.9
Ataxia 2–3 + HIT	100	61.1

*^a^Ataxia 1 walking independently*.

*^b^Ataxia 2 cannot walk without support*.

*^c^Ataxia 3 falling at upright posture*.

When the grade of ataxia and the final diagnosis of the patients are considered, it can be observed (Table 4) that none of the patients with peripheral lesion presented with grade 3 ataxia, while the absence of ataxia is not to be expected in patients with central lesions. Meanwhile, there is an overlapping of central and peripheral lesions when grade 2 ataxia is considered. Only three patients with stroke had grade 1 ataxia, in those cases, the presence of direction-changing nystagmus, positive skew, or normal HIT and Babinski’s asynergy sign guided the central etiology (Table 3).

**Table 4:** **Ataxia grade and final diagnosis**.

	Peripheral (# of patients/%)	Central (# of patients/%)
0	5/6.9	–
1	39/54.2	3/7.1
2	28/38.9	11/26.2
3	–	28/66.7

Table 3 shows the results obtained for sensitivity and specificity when combining the evaluated signs. The HINTS protocol is the one that achieves the best results (sensitivity 100%, specificity 94.4%). Associating ataxia 2–3 to other signs does not improve specificity, but it increases sensitivity to 100%. Table 5 details the sensitivities and specificities of the signs in isolation or in combination, discriminated according to the affected arterial territory. The combination of signs shows good sensitivity regardless of the affected area. On the other hand, if the signs are considered in isolation, grade 2–3 ataxia is better to diagnose AICA territory compromise, while HIT is best to diagnose PICA territory compromise. Another finding worth mentioning is that the severity of ataxia in central lesions was not statistically correlated with the patients’ age (*p* > 0.05). The caloric tests performed did not provide additional information, as their results coincide with the HIT results. The exploration of Babinski’s asynergy sign contributed to specificity in AICA, while hearing evaluation did not contribute to it.

**Table 5:** **Sign sensitivity according to the affected arterial territory**.

	AICA sensitivity (%)	PICA sensitivity (%)
Direction-changing nystagmus	60	56.3
Skew	60	53.1
HIT	30	100
Grade ataxia 1	0	9.4
Ataxia 2	20	28.1
Ataxia 3	80	62.5
Ataxia 2–3	100	90.6
Asynergy	100	90.6
HINTS	100	100
Ataxia 2–3 + nystagmus	100	100
Ataxia 2–3 + skew	100	100
Ataxia 2–3 + HIT	100	100

## Discussion

Although there are no studies that directly assess the incidence of AVS in the emergency room, it is estimated to represent 10–20% of dizziness visits (11). Of these, 25% are estimated to be due to a central vascular lesion (2). Most patients who suffer from vascular dizziness/vertigo end up having an AVS diagnosis, but according to the information available, only ~20% of them show focal neurological deficit, the rest is shown as an isolated AVS (2, 12). We found 9.36% of AVS in the group of dizziness/vertigo visits. Out of the AVS total, 36.84% presented with central vascular lesion as its cause. Although these data are not totally similar to that reported in the literature (2, 11, 12), we cannot rule out bias in the choice of patients.

This work did not find differences between groups (vestibular neuritis vs. stroke) with regard to sex. The findings that groups did not differ with sex but did differ in age and risk factors agree with other authors (2, 13) who report that patients with vascular lesions as a result of AVS are usually older and show at least one vascular risk factor.

Direction-changing nystagmus is a marker of a central lesion (1–3, 14). Therefore, no patient with vestibular neuritis presents with it, while a unidirectional nystagmus is not exclusive to peripheral vestibular lesions (14–18), as shown by the existence of patients with AICA territory lesions with these findings. Simultaneous AICA/PICA strokes may also have a unilaterally abnormal h-HIT (16), and thus its presence does not help in the differential diagnosis of central and peripheral lesions. The presence of direction-changing nystagmus shows a 57% sensitivity and a 100% specificity, values that are a bit different from those provided by other authors (12), thus we can say that its origin is almost never peripheral.

A positive HIT is a marker of a peripheral vestibular pathway lesion, so we can expect to find it in pathologies, such as vestibular neuritis. It was positive in all of our patients with neuritis, but it can also be present in central lesions with vestibular nuclei compromise (19–23), in our case we found it in AICA territory lesions (7/10), and it did not help in the differential diagnosis.

### HINTS Plus

In 2013, HINTS plus hearing loss due to cochlear or brainstem ischemia was introduced as a new sign that could potentially increase diagnostic accuracy (14, 17). The vascular supply to the inner ear, the fourth-nerve complex (i.e., cochlear, superior and inferior vestibular, and facial nerves) in the cerebellopontine angle, the cochlear nerve root entry, and the cochlear nucleus derives frequently from AICA (~80% of cases), the basilar artery (~15–20%), and only rarely *via* PICA. Hearing loss was present in only 4 of the 10 patients with AICA territory compromise. This number is not significant enough to make a differential diagnosis.

HIT with an 83% sensitivity and 100% specificity in our sample is similar to that found in the literature (1, 2, 12), since these values were obtained when considering stroke in general (AICA and PICA). If these territories are considered separately, sensitivity is 100% in the case of PICA, but only 30% in AICA, also coinciding with what has been reported in the past (12). In patients with AVS, a negative HIT indicates a central lesion, but a positive one does not rule out a central lesion, mainly in patients with lesions in the AICA territory.

The presence of Skew deviation described in central lesions (7, 8) may be found in patients with vestibular neuritis (4/72) and even be absent in central lesions (PICA 15/32 and AICA 4/10) and therefore, should not be used as an isolated sign. The finding of Skew deviation by means of the cover cross-cover test showed 54.8% sensitivity and 94.4% specificity, which is slightly more sensitive than the one previously reported, and with a similar specificity (2, 12, 18). Its absence or presence does not allow us to differentiate between central or peripheral lesions, though its presence in peripheral lesions is uncommon and of lesser magnitude.

Truncal ataxia was chosen as a test since it is an easy to evaluate sign, even for physicians without specific training in clinical neurology. Also, several authors mention truncal ataxia as a valuable element in the differential diagnosis of AVS (3, 10). Though it is a well-established scientific fact that the findings of the HINTS protocol are solid and have been replicated by several groups (2, 14, 20, 24), many clinical neurologists consider that the almost constant presence of significant grade ataxia has been undervalued. When exploring the presence and severity of ataxia in our sample, we found that a grade 2–3 ataxia has 92.9% sensitivity and 61.1% specificity values, which we cannot compare with other works as we found no trial that has evaluated its diagnostic accuracy. We also found that all the patients with central lesion had some degree of ataxia, and none of them was free from this sign; thus, we can say that the absence of ataxia in the clinical picture predicts a peripheral lesion. As regards grade 3 ataxia (66.7% sensitivity and 100% specificity), none of the patients with a peripheral lesion had grade 3 ataxia (100% specificity), a finding similar to that reported in the literature (2). This allows us to say with a high degree of certainty that a person with grade 3 ataxia has a central lesion as cause of his/her AVS, and thus, it is not necessary to look for other neurological or otoneurological signs. Babinski’s asynergy (9) sign was particularly useful as it made it possible to evaluate the patient while in bed, instead of asking him/her to walk, and it was present in 100% of the patients with grade 3 ataxia and in all patients with stroke and grade 1 ataxia.

Another element to consider is the difficulty one faces when applying the HINTS protocol in an emergency room. Though in the case of subspecialists there seems to be a good reproducibility of the explored signs (2), in many occasions, there are doubts about this when it is the physician on duty who has to apply this protocol, as they may lack the appropriate training on otoneurological examination. In 2015, Kerber et al. (25) reported their experience with HINTS in a large cohort from the University of Michigan in Ann Arbor. In their series, they had only one false negative stroke MRI. Potential limitations of this study include mixed-skill rater examiners and only fair inter-rater agreement (Kappa: 0.24–0.40).

When we applied the HINTS protocol in our sample, we obtained a 100% sensitivity and a 94.4% specificity, values similar to those found in the original paper (100% sensitivity and 96% specificity) (2). From an educational point of view, it is our experience that the clinical training of an intern presents little difficulty during the first year of internship and is highly difficult by the end of second year. This is based on 3 neurology interns per year in our hospital, with 96 days on active call and an average of 10 admissions per shift: 960 acute patients per year.

Although ataxia evaluation and staging is relatively easy and its sensitivity is not negligible, if the idea is to use this opportunity to detect a vascular lesion early, it is not enough to recommend its use in isolation in the emergency room. However, when combining grade 2–3 ataxia with other signs already evaluated in the HINTS protocol, such as skew deviation, direction-changing nystagmus, or HIT, sensitivity shows great improvement and achieves 100%, though specificity does not (61.1%). In our case, we chose a combination of grade 2–3 ataxia and nystagmus, as we consider that both elements can be easily recognized and interpreted by physicians with a minimum knowledge of otoneurology.

Although it is true that we improved sensitivity, specificity did not improve, and it was even lower than that obtained by applying the HINTS protocol. However, if we consider that the idea is to detect patients with a probable central lesion, its excellent sensitivity is enough for it to be used in an emergency room, where what counts is being able to separate VN from stroke and avoid wasting resources. Clinicians should evaluate patients with AVS so as to replicate the data found in this work, trying to overcome the limitations we have found.

### Limitations

The findings of grade 3 ataxia and positive asynergy in patients with AICA infarction should be viewed with caution due to the small number of cases (*n* = 10).

## Conclusion

Our results confirm the high effectiveness of the HINTS protocol in the differential diagnosis of stroke in patients with AVS. In addition, a simple sign, such as ataxia staging, is highly sensitive to avoid false negatives in patients with AVS.

## Author Contributions

SC directed the investigation. CM, GZ, and MM recruited the patients, examined them, and reported the data. AB-C contributed with his personal experience with HINTS protocol. LL contributed with his personal experience with AVS in the ER. Finally, CG made critical revisions to the manuscript.

## Conflict of Interest Statement

The authors declare that the research was conducted in the absence of any commercial or financial relationships that could be construed as a potential conflict of interest. The reviewer JH and handling Editor declared their shared affiliation, and the handling Editor states that the process nevertheless met the standards of a fair and objective review.
